# Reading Books Helps Children in Poverty Become More Resilient: Results From a Population-based Longitudinal Study in Japan

**DOI:** 10.2188/jea.JE20240329

**Published:** 2026-02-05

**Authors:** Yukako Tani, Aya Isumi, Yui Yamaoka, Takeo Fujiwara

**Affiliations:** 1 Department of Public Health, Institute of Science Tokyo, Tokyo, Japan; 2 Department of Health Policy, Institute of Science Tokyo, Tokyo, Japan; 3 Department of International Health, Bloomberg School of Public Health, Johns Hopkins University, Baltimore, MD, USA

**Keywords:** reading books, poverty, resilience, children

## Abstract

**Background:**

Resilience is an important ability in reducing subsequent health risks from poverty. This study aimed to examine whether reading books in fourth grade boosts resilience in sixth grade and whether poverty status modifies the association.

**Methods:**

We used a part of longitudinal data from 2018 to 2020 from the Adachi Child Health Impact of Living Difficulty (A-CHILD) study. In this analysis, we used all fourth-grade elementary school students and their caregivers’ follow-up data (*n* = 3,136 9- to 10-year-olds; 49.6% boys; follow-up rate: 87%). Poverty and the number of books read in fourth grade were assessed at baseline. Child resilience in fourth and sixth grade was assessed by caregivers using the Children’s Resilient Coping Scale.

**Results:**

In fourth grade, 20% of children read no books, while 15% read 4 or more books per week. Children who read more books at baseline became more resilient in sixth grade, even after adjustment of resilience in fourth grade. Poverty in fourth grade was associated with lower resilience in both fourth and sixth grade; however, when stratified by poverty status, the number of books read was significantly associated with higher resilience only among children in poverty (eg, coefficient 5.13; 95% confidence interval CI, 1.20–9.06 for ≥4 books vs none).

**Conclusion:**

For elementary school children in Japan, reading books boosts resilience, especially among children in poverty. Educational policy on reading books during elementary school may be important to address child poverty.

## INTRODUCTION

Child poverty remains a major problem worldwide, with an estimated 1 in 7 children living in poverty even in developed countries, including Japan.^1^^,^^2^ Poverty is one of the adversity experiences of childhood, and experiencing poverty is a powerful predictor of adverse outcomes in both physical and mental health over the subsequent life course.^3^^,^^4^ As direct measures against child poverty, such as conditional or non-conditional cash transfer,^5^ require a huge budget and are not easy to implement, it is needed to focus on protective factors for child health and development among children with poverty and to implement interventions increasing protective factors.

Resilience is one of the protective factors for physical and mental health development by mitigating the toxic effects of childhood adversity. Although there is no universal definition of resilience, it is generally understood as the capacity of a system to adapt successfully to challenges that threaten the function, survival, or future development of the system.^6^ Resilience has been reported to mitigate child health risks due to poverty. For example, in a study of children in the United States, resilience moderated the association between low socioeconomic status (SES) and higher body mass index.^7^ In a study of Canadian youth, resilience was associated with lower levels of asthma-related inflammation in low-SES children.^8^ In a study of children and youth in the United States, resilience moderated the association between adverse childhood experiences, including economic hardship, and depression.^9^ Therefore, it is important to find the factors that develop resilience for children in poverty.

One possible feasible and economical intervention that develops resilience in children of early adolescence in poverty can be promotion of reading books. Previous intervention studies for toddlers have shown that reading books has substantial benefits for psychological development by providing cognitive stimulation, especially in developing countries^10^ and poor areas in developed countries.^11^ Children in poverty are at higher risk for lower levels of cognitive ability and self-regulation skills,^12^ which can be reinforced by reading books.^13^

The association between reading and resilience may vary depending on whether the child is in poverty; in other words, there may be an interaction between reading books and poverty. One possible reason for the interaction between poverty and reading books is the differential impact of reading books on executive function, which is an important factor for the development of resilience.^14^ The act of reading books requires executive functions, such as working memory (eg, the ability to read a book while remembering information about the previous context and characters), self-control (eg, the ability to suppress external stimuli and concentrate on reading), and cognitive flexibility (eg, the ability to accept new perspectives and modify understanding when interpreting metaphors and abstract expressions), but in the case of poverty, poor executive function have been observed.^15^ Therefore, despite their low executive function, the habit of reading books continuously may help children in poverty develop executive function, and thus resilience, more than children in non-poverty. Other reasons for the interaction between poverty and reading books may be the stress-reducing of reading.^16^^,^^17^ Chronic strains, such as hunger and unsafe living conditions due to poverty, are associated with lower resilience.^18^^,^^19^ Children in poverty who are under high stress may reap more of the stress-reducing benefits of reading books than children in non-poverty, and as a result, may develop more resilience. Therefore, we hypothesize that there is an interaction between poverty and reading books on the association with resilience.

In addition, it is necessary to check whether reading books boosts resilience in general or specific to children with poverty because a universal provision of health policy on reading books might widen the disparity among children.^20^ Alternatively, if reading books is more effective in increasing resilience, especially among children in poverty, it would be acceptable to implement a health policy to read more books in general, which is beneficial to avoid stigmatization.

Furthermore, the association between the number of books read and resilience is an important consideration. In a 2018 survey of elementary school students in Japan, the average number of books read was 2.5 books per week, and half of the children read none.^21^ In this nationwide study, the number of books read was trending downward, averaging four books per month in junior high school and one book per month in high school.^21^ In the 2018 Organisation for Economic Co-operation and Development survey, half of the 15-year-olds agreed that “I read only to get information that I need”, a prevalence that had increased since 2009.^22^ Several studies have shown that the greater number of books available during childhood or adolescence is associated with higher later literacy and cognitive performance,^23^^,^^24^ while evidence on reading book contents, such as genres, is limited and results on the association with cognitive performance are mixed.^25^^,^^26^ We hypothesized that frequent reading, or habitual exposure to book reading, might be important for the development of resilience through the development of executive function and stress reduction. Therefore, we decided to evaluate the association between the number of books read and resilience.

This study aimed first to determine the association of the number of books read with a child’s resilience. Second, it aimed to examine whether the association between reading books and resilience differs depending on whether the child is in poverty or non-poverty.

## METHODS

### Study design and participants

This study used data from the Adachi Child Health Impact of Living Difficulty (A-CHILD) project, which was established in 2015 to evaluate the determinants of health among children in Adachi, Tokyo, Japan (see protocol paper^27^). In this study, longitudinal data collected with fourth-grade students (aged 9 to 10 years) in October 2018 and then followed through sixth grade (aged 11 to 12 years) in October 2020 was used (follow-up time: 2 years). The survey covered children and their caregivers in all 69 public elementary schools in Adachi. Self-report questionnaires with anonymous unique IDs were distributed to 5,311 elementary school students in the fourth grade (aged 9 to 10 years) and their caregivers. A total of 4,605 child-caregiver pairs completed questionnaires (response rate: 86.7%). Of these respondents, 4,290 pairs provided informed consent and completed all questionnaires. Eighty-seven percent of participants completed follow-up self-reported questionnaires in 2020 (*n* = 3,733). For the analysis, 3,136 participants were included after those who did not complete the questions related to child resilience (*n* = 7), reading books (*n* = 89), or poverty status (*n* = 501) were excluded. A flow chart of the participants is presented in eFigure 1. The A-CHILD protocol and use of the data for this study were approved by the Ethics Committee at the Tokyo Medical and Dental University (No. M2016-284).

### Resilience

Child resilience was assessed using the Children’s Resilient Coping Scale (CRCS) through a caregiver questionnaire. This scale was developed by Japanese experts to suit the Japanese context and has high internal consistency (Cronbach’s alpha: 0.80) and sufficient validity.^28^ CRCS consists of the following eight items: 1) speaks positively about their future; 2) tries to do their best; 3) able to tolerate teasing or mean comments well; 4) knows how to properly greet others; 5) able to get ready for school, study, and do their chores without directions; 6) seeks appropriate advice when necessary; 7) able to give up things they want or do things that they do not like to do for better future outcomes; and 8) able to ask questions to learn about what they do not understand. Caregivers rated their child’s resilience on a scale of 0 (never) to 4 (very frequently). The total score for eight items was rescaled to range from 0 to 100, with higher scores indicating higher resilience. Cronbach’s α for the eight items was 0.84 in this study sample.

### Book reading

Children’s book reading was assessed using the following question: “How many books (excluding comics and magazines) do you read in a month?” The options were: rarely read, about 1 book, 2–3 books, 4–10 books, 11–15 books, and more than 16 books. To make it easier to understand the number of habitual books read, each option was assigned to the following categories: 0, 0.25, 0.5–0.75, 1–2.5, 2.75–3.75, and 4 or more books per week, respectively, and these were further divided into four categories: none, <1 book, 1–3 books, and ≥4 books per weeks.

### Poverty

Poverty was assessed at baseline through questionnaires completed by caregivers. In this study, following previous research,^29^^–^^31^ poverty was defined based on three criteria: (1) annual household income <JPY 3.0 million, (2) lack of materials or household goods needed by the child. (3) have problems paying for lifeline utilities. If at least one of these three criteria was met, the household was defined as poverty. Poverty by this definition has predictive validity because it is associated with child mental health, dental caries, and other problems.^29^^–^^31^ Regarding material deprivation, the caregivers were asked whether their family does not have any of the following items due to financial reasons: 1) age-appropriate books (ie, children’s picture books); 2) sporting goods, stuffed animals, and toys for children; 3) room or space where the child can do homework at home; 4) washing machine; 5) rice cooker; 6) vacuum cleaner; 7) heater; 8) air conditioner; 9) microwave; 10) phone (including landline and mobile); 11) bathtub, 12) bed or futon for each family member; 13) savings for an emergency (50,000 yen or more), or 14) none.^29^ Regarding problems with lifeline payments, caregivers were asked if the respondent’s family had experienced in the previous year an inability to pay for financial reasons: 1) fees for school field trips; 2) fees or transportation costs for extracurricular classes; 3) school lunch fees; 4) rent; 5) mortgage; 6) electric utility bill; 7) gas utility bill; 8) water and sewerage bill; 9) phone bill (including landline and mobile); 10) insurance bill, including public pension, health insurance, and long-term care insurance; 11) commuting costs, or 12) none.^29^

### Covariates

Household and caregiver status were assessed using the caregiver-report questionnaire in fourth grade. Household status included cohabitation with the child’s parents and grandparents and the presence of siblings. The caregiver’s status included the responding caregiver’s mental health, mother’s age, father’s age, mother’s educational attainment, father’s educational attainment, mother’s employment status, and father’s employment status. The child’s status included sex and baseline resilience. The caregiver’s mental health was assessed using the Kessler 6 scale (Japanese version),^32^ and higher scores indicate more frequent problems with psychological distress.^33^ Parental educational attainment was categorized into three groups (low: junior high school, dropped out of high school, or high school; middle; professional school, some college, or dropped out of college; and high: college or higher).^34^

### Statistical analysis

First, the characteristics of children and their caregivers were stratified by reading book categories, and the differences in characteristics were tested using Chi-square tests or analysis of variance. Second, to examine the interaction effect of the number of books read and poverty on a child’s resilience, a multivariate linear regression model was performed. Models were selected as follows: model 1 adjusted for potential confounders; model 2 additionally adjusted for the baseline child’s resilience. Third, the data were analyzed with stratification by poverty status to show how much reading is needed by children in poverty. Participants with missing data on the covariates were included in the analysis. All analyses were conducted using Stata statistical software Macro Package version 17 (Stata Corporation, College Station, TX, USA).

## RESULTS

The characteristics of the participants at baseline are shown in Table 1. By our definition of poverty, 20% of children’s families were poor. About 80% of the children lived only with their parents, and less than 10% were in three-generation families. Eighty percent of the children had siblings. Half of the children were girls. More than half of the children read less than one book per week. On the other hand, 15% of the children read four or more books per week. Children tended to read more if they were girls, non-poor, had only younger siblings, the mother and father had a higher educational background, and the mother worked full-time.

**Table 1.** Characteristics of participants in Japanese school children (*n* = 3,136)

	Total		Number of books read (/week)				*P*-value
			none	<1 book	1–3 books	≥4 books	
			(*n* = 468, 19.5%)	(*n* = 1,073, 34.2%)	(*n* = 984, 31.3%)	(*n* = 468, 14.9%)	

	*n*	%	%	%	%	%	
Household status							
Poverty status							
Non-poverty	2,516	80.2	78.1	78.4	82.7	82.1	0.03
Poverty	620	19.8	21.9	21.6	17.3	17.9	
Cohabitation status							
Parents	2,569	81.9	80.0	82.1	82.5	82.7	0.11
Parents and grandparent (s)	239	7.6	7.7	7.5	8.0	7.1	
Single parent	236	7.5	10.0	7.0	6.4	7.9	
Single parent and grandparent (s)	68	2.2	1.8	2.8	2.3	0.9	
Other	24	0.8	0.5	0.7	0.7	1.5	
Child’s siblings							
No	621	19.8	17.5	20.2	20.9	19.4	<0.001
Only older sibling(s)	1,130	36.0	41.6	38.4	33.1	29.5	
Only younger sibling(s)	1,036	33.0	28.8	30.1	35.3	40.6	
Both older and younger sibling(s)	349	11.1	12.1	11.3	10.7	10.5	
Caregiver’s status							
Respondent’s K6							
<5	2,087	66.5	64.3	66.2	68.7	65.8	0.37
≥5	976	31.1	33.9	31.5	28.6	32.1	
Missing	73	2.3	1.8	2.3	2.7	2.1	
Mother’s age, years							
<35	1,055	33.6	34.4	32.7	33.4	35.3	0.25
35–44	1,174	37.4	34.2	38.5	38.1	37.8	
≥45	857	27.3	29.8	26.5	27.4	25.9	
Missing	50	1.6	1.6	2.3	1.0	1.1	
Father’s age, years							
<35	665	21.2	23.9	22.1	19.7	18.8	0.02
35–44	1,000	31.9	27.8	33.3	31.2	35.5	
≥45	1,205	38.4	39.0	35.5	41.9	37.2	
Missing	266	8.5	9.3	9.1	7.2	8.5	
Mother’s education							
Low	871	27.8	29.8	32.2	24.2	22.6	<0.001
Middle	1,161	37.0	36.3	35.1	40.3	35.3	
High	588	18.8	15.7	16.5	21.3	22.4	
Other/missing	516	16.5	18.2	16.2	14.1	19.7	
Father’s education							
Low	956	30.5	35.2	32.6	27.5	25.6	<0.001
Middle	488	15.6	13.7	16.9	15.2	15.6	
High	1,077	34.3	30.9	30.2	39.8	36.8	
Other/missing	615	19.6	20.1	20.3	17.4	22	
Mother’s employment status							
Full-time	729	23.2	20.8	21.5	23.3	30.3	0.01
Part-time	1,472	46.9	50.4	48.3	47.0	39.3	
Self-employed/side work/other	222	7.1	7.7	6.5	6.8	8.1	
Not employed	665	21.2	19.3	21.9	21.7	20.9	
Missing	48	1.5	1.8	1.8	1.2	1.3	
Father’s employment status							
Full-time	2,336	74.5	70.5	74.7	76.2	75.6	0.20
Part-time	54	1.7	1.1	1.8	1.8	2.1	
Self-employed/side work/other	448	14.3	16.7	14.3	13.2	13.5	
Not employed	27	0.9	1.6	0.5	0.9	0.6	
Missing	271	8.6	10	8.9	7.8	8.1	
Child’s status							
Sex							
Boy	1,556	49.6	58.1	52.2	42.7	47.2	<0.001
Girl	1,580	50.4	41.9	47.8	57.3	52.8	
CRCS score (0–100)	Mean	SD	Mean	Mean	Mean	Mean	
Fourth grade (baseline)	69.8	16.1	66.7	69.3	71.6	71.2	<0.001
Sixth grade	71.6	16.2	69.1	71.0	73.0	73.4	<0.001

CRCS, Children’s Resilient Coping Scale; K6, Kesseler 6-item Psychological Distress Scale; SD, standard deviation.

The *P*-value for a chi-squared test or analysis of variance.

The association between the number of books read and resilience is shown in Table 2. Multiple linear regression analysis revealed that a high number of books read was associated with higher resilience (coefficient 3.70; 95% CI 1.76–5.64) after adjusting potential confounders. After adjusting for baseline resilience, compared to children who did not read, those who read four or more books a week were 1.78 points higher in resilience (95% CI, 0.19–3.37). When poverty status and an interaction term between the number of books read and poverty status were added to the model, there was no main effect of reading books, but there was a main effect of poverty (coefficient −3.09; *P* = 0.02) and an interaction effect for reading four or more books (*P* = 0.029; model 4).

**Table 2.** Associations between the number of books read, poverty, and resilience among elementary school children (*n* = 3,136)

	Model 1	Model 2	Model 3	Model 4
	coefficient (95% CI)	coefficient (95% CI)	coefficient (95% CI)	coefficient (95% CI)
Number of books read (/week)				
None	ref	ref	ref	ref
<1 book	**1.77 (0.18–3.36)**	0.69 (−0.61–1.99)	0.70 (−0.60–1.99)	0.70 (−0.76–2.16)
1–3 books	**3.12 (1.49–4.75)**	1.02 (−0.32–2.35)	0.99 (−0.35–2.32)	0.86 (−0.63–2.35)
≥4 books	**3.70 (1.76–5.64)**	**1.78 (0.19–3.37)**	**1.76 (0.17–3.34)**	0.92 (−0.85–2.69)
Poverty status				
Non-poverty		-		
Poverty			**−2.38 (−3.66–−1.11)**	**−3.09 (−5.64–−0.54)**
Number of books read × Poverty status				
<1 books × Poverty		-	-	−0.05 (−3.18–3.08)
1–3 books × Poverty		-	-	0.43 (−2.86–3.72)
≥4 books × Poverty		-	-	**4.41 (0.45–8.37)**

CI, confidence interval.

Model 1: Adjusted for number of books read, child’s sex, siblings, father’s age, mother’s and father’s education, and mother’s employment status.

Model 2: model 1 + adjusted for baseline child’s resilience.

Model 3: model 2 + adjusted for baseline poverty status.

Model 4: model 3 + adjusted for interaction terms of number of books read and poverty status.

Table 3 shows the association between the number of books read and resilience according to poverty status. When adjusting potential confounders, children in non-poverty were more resilient if they read books (Table 3). However, when baseline resilience was added, the association between books and resilience was no longer significant. In contrast, children in poverty were more resilient if they read four or more books, and the association was significant when baseline resilience was added.

**Table 3.** Associations between number of books read and resilience according to poverty among elementary school children (*n* = 3,136)

	Number of books read (/week)	Model 1	Model 2
		coefficient (95% CI)	coefficient (95% CI)
Non-poverty *N* = 2,516	None	ref	ref
	<1 book	**2.01 (0.26–3.77)**	0.69 (−0.75–2.12)
	1–3 books	**3.22 (1.44–5.01)**	0.77 (−0.69–2.23)
	≥4 books	**2.77 (0.65–4.89)**	0.81 (−0.92–2.55)
Poverty *N* = 620	None	ref	ref
	<1 book	0.85 (−2.87–4.57)	0.58 (−2.49–3.66)
	1–3 books	2.17 (−1.79–6.12)	1.73 (−1.53–4.98)
	≥4 books	**7.21 (2.44–11.98)**	**5.13 (1.20–9.06)**

CI, confidence interval.

Model 1: Adjusted for number of books read, child’s sex, siblings, father’s age, mother’s and father’s education, and mother’s employment status.

Model 2: Model 1 + adjusted for baseline resilience.

**Figure 1.**
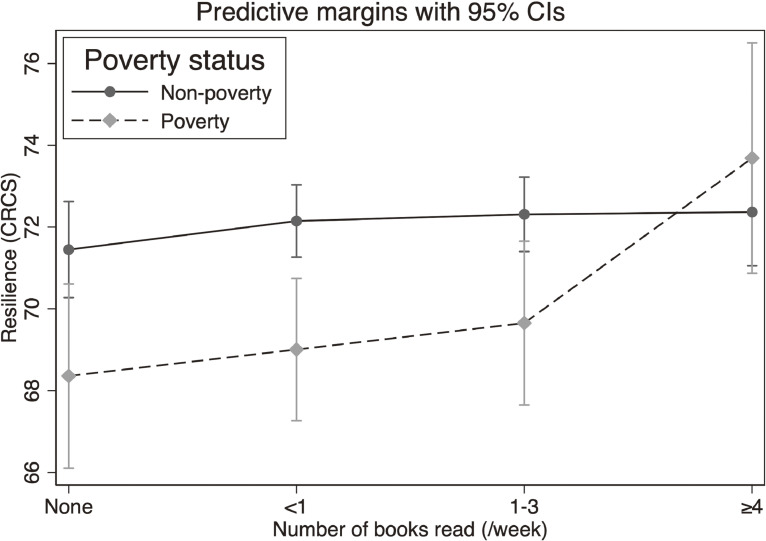
Associations between the number of books read and resilience according to poverty among elementary school children. Models were adjusted for the child’s sex, siblings, father’s age, mother’s and father’s education, mother’s employment status, and child’s baseline resilience. CI, confidence interval; CRCS, Children’s Resilient Coping Scale.

The results, adjusting for potential confounders and baseline resilience, are shown in Figure 1. Children in poverty were less resilient than non-poverty children if they read three or fewer books. However, for children who read four or more books, resilience was higher for children in poverty and equal to or higher than that of non-poverty children.

## DISCUSSION

The number of books read in fourth grade was found to be associated with a higher resilience among at sixth grade in elementary school children. Reading books moderated the association between poverty and child resilience. Being in poverty was associated with low child resilience, but children who read four or more books per week were as resilient as non-poverty children.

Reading books was positively associated with resilience among elementary school children. To the best of our knowledge, no previous studies have examined the association between the number of books read and resilience using a longitudinal design. Nonetheless, the present study is in line with studies that assessed the home library size and problem-solving skills.^23^^,^^35^ Those who had more books in their homes at age 16 years had better problem-solving skills later in life.^23^ Our study did not examine where children obtain their books, so future research will need to investigate sources of books, such as home, school, and library.

Children in poverty were less resilient, but reading books moderated this association. Reading books did not mediate the association between poverty and resilience (eTable 1); in other words, poverty did not prevent children from reading adequate books. Of the 620 children in poverty, 84 (14%) who read four or more books were highly resilient after a 2-year follow-up, comparable to non-poor children. There are several possible reasons why reading books benefits resilience among children in poverty. Children in poverty face more problems related to poverty, but they may learn problem-solving skills through reading books.^23^ Reading books written by or regarding people who have experienced poverty may help children in poverty to become aware of their situation and how to overcome it by nurturing their potential resilience. Past mastery experiences and being able to learn from other people’s behavior have been reported to be important for youth resilience.^36^ Children in poverty could learn from the experiences and behaviors of the characters in the book.

We found that reading four or more books per week was associated with higher resilience among children in poverty. In a study of Japanese elementary school students, the amount of books read assessed by the number of books checked out from the library in the third grade predicted improvement in reading comprehension 1 year later.^26^ Among children in poverty, access to cognitively enriching activities and the quantity and complexity of language input are deprived.^15^ Therefore, habitually reading many books may have contributed to resilience development through the development of executive functions. Another possible mechanism is stress reduction through reading. In a study of college students, an intervention of 30 minutes of reading articles taken from various Internet sites, including historical events and innovative technologies, for 3 weeks has been reported to reduce stress.^16^ It may suggest that reading a certain number of books on an ongoing basis, regardless of genre, may reduce stress and increase resilience. Another mechanism is the development of emotional regulation skills through reading books. Childhood poverty has been reported to be associated with reduced emotional regulation ability.^37^ A recent systematic review reported that reading practices have positive impacts on emotion regulation, empathy, and theory of mind.^38^ Reading practices may have developed emotional regulation skills, which are important for the development of resilience,^6^ by putting oneself in the perspective of the characters and imagining their emotions as they read. Further research is needed to examine the mechanism of the relationship between reading and resilience.

For both poor and non-poor status, the benefit of resilience from reading books decreased when adjusted for baseline resilience. This is likely because children with higher baseline resilience read more books, which is a confounding factor. On the other hand, the adjustment for baseline resilience may be an over-adjustment because it may be a mediator between reading habits up to fourth-grade and sixth-grade resilience. More detailed mediation analysis or randomized controlled studies of the efficacy of reading books in fourth grade on the development of resilience are warranted.

This study has several limitations. First, we lacked data on the genre of books being read, which may have influenced the effect of reading books on resilience. In a recent survey of Japanese elementary school students, mystery illustrated books, natural science, and fantasy topped the list of favorite book genres, excluding comics.^39^ Evidence on the genre of books being read was mixed, with fiction positively correlating with reading comprehension in a study of American sixth grade children,^25^ and nonfiction, but not fiction, correlating with reading comprehension in a study of Japanese third- and fourth-grade children.^26^ Given that fiction has the potential to strengthen readers’ empathy and emotional regulation by helping them understand other people’s perspectives, or that immersing themselves in a story can free them from the stresses of reality and increase their resilience, while nonfiction may help them overcome adversity, we will need to examine the genre of reading. Furthermore, volume per book has not been assessed. It is easy to read four books a week with a few pages, but difficult to read more than four books a week with many pages. Future research is needed to determine what genre of books and how much volume per book is effective for resilience in poverty. Second, this study did not assess the accessibility to books, and there is no information on where children obtain their books and where they read them. For interventions with children in poverty, it would be necessary to examine how books are obtained. Future research should examine the use of school libraries and community libraries, which may be more accessible to children in poverty. Third, there may be response bias because children’s resilience is assessed by their parents. A recent review of assessments of children’s resilience found that in most studies, the reporters were either the children themselves or their parents, and bias due to differences in reporters was not examined.^40^ In this study, compared to responses by mothers, those by fathers tended to rate their children’s resilience lower. The difference in whether father/mother responded to child resilience was not a confounding factor because it was not related to the number of books read. However, there may be response bias concerning children’s resilience. Future research should examine bias due to differences in resilience respondents. Fourth, there is the presence of unmeasured confounding factors. When baseline characteristics were examined in children in poverty, none were significantly associated with the number of books read (eTable 2). Future studies will need to examine lifestyle habits other than reading that may be confounding factors. Finally, about 500 children with missing information about poverty were excluded from the analysis. In addition, it is possible that participants of lower economic status (ie, poverty) dropped out during the follow-up, which may have underestimated the results. However, since all elementary schools in Adachi city were included and the follow-up rate was 87%, some generalizability to other locations of the country may be preserved.

In conclusion, the number of books read in fourth grade was associated with higher resilience in sixth-grade children. Reading books mitigated lower resilience due to poverty. Children in poverty reading four or more books per week had high resilience comparable to that of non-poor children. Because reading books did not mediate the association between poverty and resilience, it would be better to provide children in poverty the opportunity to access enough books than to provide financial support to families facing poverty. Creating an environment that encourages poor children to read more books may, which can be done by schools or communities, be effective in preventing the subsequent negative effects of poverty.

## SUPPLEMENTARY MATERIAL

eTable 1. Mediation analysis between poverty and child’s resilience in Japanese school children (n = 3,136)

eTable 2. Characteristics of participants in Japanese school children in poverty (n = 620)

eFigure 1. The participants flow for the analytic sample
