# Supplementary material for: Reading Books Helps Children in Poverty Become More Resilient: Results From a Population-based Longitudinal Study in Japan
Source: J Epidemiol. 2026 Feb 5;36(2):51–7. doi: 10.2188/jea.JE20240329 (PMC12783505; doi:10.2188/jea.JE20240329)
Supplement: Supplementary file 1 [file je-36-051-s001.pdf]

eTable 1. Mediation analysis between poverty and child's resilience in Japanese school children (n=3,136)

Mediator	Estimated Indirect Effect coefficient (95% CI)	*P* value
Number of books read	-0.12 (-0.33 to 0.09)	0.25

CI, confidence interval.

Mediation analysis was assessed using Stata's Paramed package.

Poverty status was treated as a binary, with 0 = non-poverty and 1 = poverty.

Models: Adjusted for child's sex, siblings, father's age, mother's and father's education, and mother's employment status.

eTable 2. Characteristics of participants in Japanese school children in poverty (n=620)

	Total		Number of books read				p-value
			none (n=134, 21.6%)	<1 book (n=232, 37.4%)	1–3 books (n=170, 27.5%)	≥4 books (n=84, 13.5%)	
	n	%	%	%	%	%	
Household status							
Poverty status							
Low income (<JPY 3 million)							
No	273	44.0	44.8	43.5	45.3	41.7	0.95
Yes	347	56.0	55.2	56.5	54.7	58.3	
Lack of materials or household goods							
No	270	43.5	45.5	39.2	46.5	46.4	0.42
Yes	350	56.5	54.5	60.8	53.5	53.6	
Paying problems for lifeline utilities							
No	422	68.1	64.2	68.1	68.2	73.8	0.53
Yes	198	31.9	35.8	31.9	31.8	26.2	
Cohabitation status							
Parents	364	58.7	59.7	58.6	59.4	56.0	0.36
Parents and grandparent (s)	40	6.5	6.0	6.0	7.6	6.0	
Single parent	169	27.3	30.6	26.3	24.1	31.0	
Single parent and grandparent (s)	36	5.8	3.0	7.3	7.6	2.4	
Other	11	1.8	0.7	1.7	1.2	4.8	
Child's siblings							
No	125	20.2	18.7	22.0	22.4	13.1	0.12
Only older sibling(s)	224	36.1	44.8	36.2	28.8	36.9	
Only younger sibling(s)	179	28.9	23.1	27.6	34.7	29.8	
Both older and younger sibling(s)	92	14.8	13.4	14.2	14.1	20.2	
Caregiver's status							
Respondent's K6							
<5	332	53.5	54.5	54.3	51.2	54.8	0.94
≥5	271	43.7	44.0	42.2	45.9	42.9	
Missing	17	2.7	1.5	3.4	2.9	2.4	
Mother's age, years							
<35	285	46.0	43.3	43.1	49.4	51.2	0.81
35–44	197	31.8	32.8	31.9	31.2	31.0	

≥45	129	20.8	22.4	22.8	18.2	17.9	
Missing	9	1.5	1.5	2.2	1.2	0.0	
Father's age, years							
<35	136	21.9	22.4	22.4	21.8	20.2	0.16
35–44	126	20.3	11.9	23.3	19.4	27.4	
≥45	173	27.9	35.8	24.1	29.4	22.6	
Missing	185	29.8	29.9	30.2	29.4	29.8	
Mother's education							
Low	234	37.7	37.3	40.5	35.3	35.7	0.28
Middle	186	30.0	31.3	27.2	33.5	28.6	
High	48	7.7	7.5	5.2	11.8	7.1	
Other/missing	152	24.5	23.9	27.2	19.4	28.6	
Father's education							
Low	218	35.2	37.3	32.8	35.3	38.1	0.38
Middle	89	14.4	17.9	11.2	15.9	14.3	
High	79	12.7	9.7	13.4	15.9	9.5	
Other/missing	234	37.7	35.1	42.7	32.9	38.1	
Mother's employment status							
Full-time	112	18.1	14.9	18.5	14.7	28.6	0.22
Part-time	352	56.8	59.7	53.0	58.2	59.5	
Self-employed/side work/other	41	6.6	6.7	7.8	7.1	2.4	
Not employed	103	16.6	16.4	18.5	18.2	8.3	
Missing	12	1.9	2.2	2.2	1.8	1.2	
Father's employment status							
Full-time	286	46.1	42.5	47.8	45.3	48.8	0.97
Part-time	29	4.7	3.0	4.7	5.3	6.0	
Self-employed/side work/other	96	15.5	17.9	15.1	15.3	13.1	
Not employed	18	2.9	4.5	2.2	2.9	2.4	
Missing	191	30.8	32.1	30.2	31.2	29.8	
Child's status							
Sex							
Boy	301	48.5	56.7	46.6	42.9	52.4	0.09
Girl	319	51.5	43.3	53.4	57.1	47.6	
Baseline resilience	Mean	SD	Mean	Mean	Mean	Mean	
CRCS score (0–100)	66.3	17.7	65.7	65.8	65.8	69.5	0.32

JPY, Japanese yen; K6, Kessler 6; SD, standard deviation.
p-value for a chi-squared test or ANOVA.

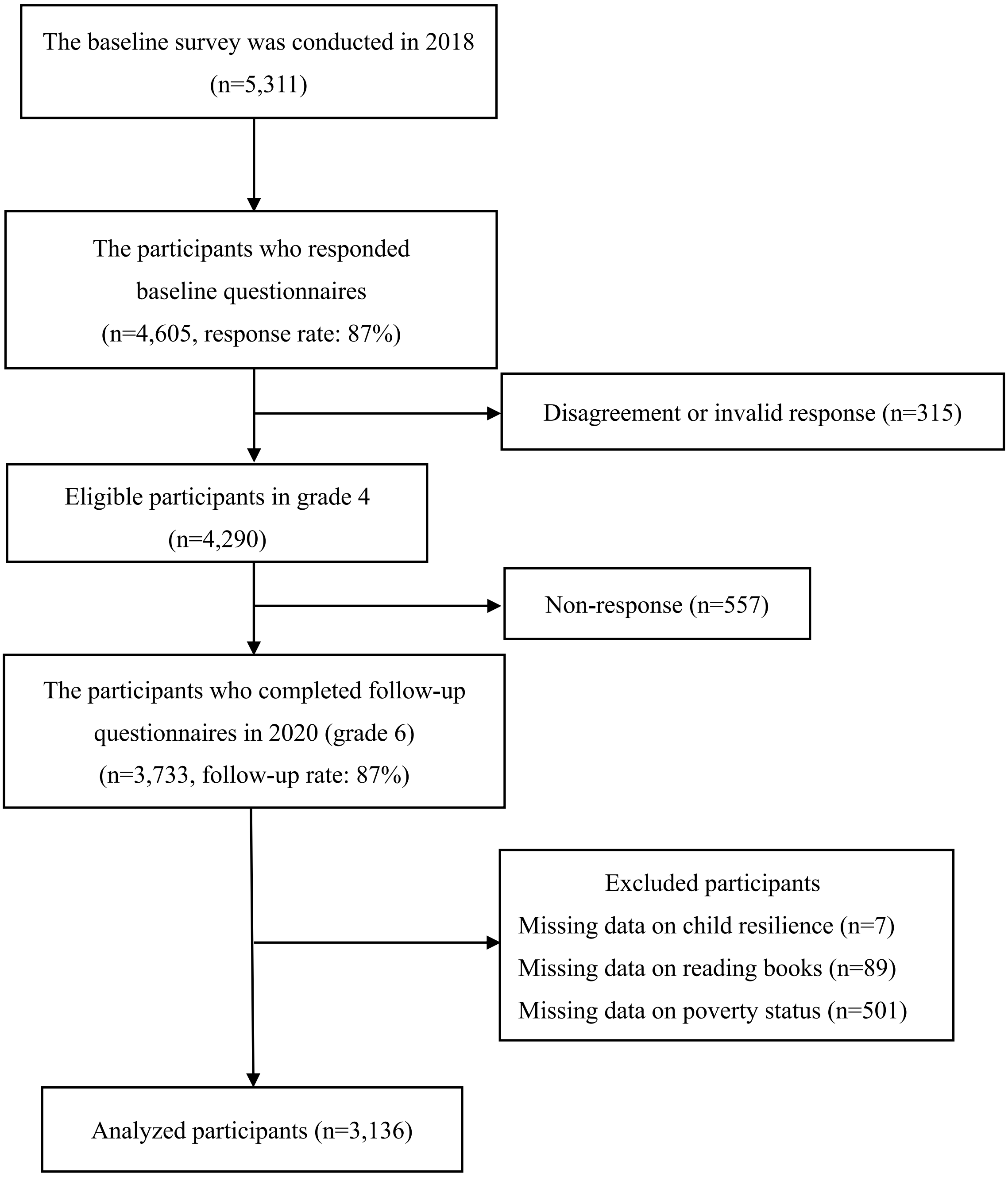

eFigure 1. The participants flow for the analytic sample
